# Up-regulated RFC2 predicts unfavorable progression in hepatocellular carcinoma

**DOI:** 10.1186/s41065-021-00179-9

**Published:** 2021-05-22

**Authors:** Zaixiong Ji, Jiaqi Li, Jianbo Wang

**Affiliations:** 1 Department of Interventional Radiology, Shanghai Jiao Tong University Affiliated Sixth People’s Hospital, No. 600, Yishan Road, Xuhui District, Shanghai, 200233 China; 2 Shanghai University of Traditional Chinese Medicine, Shanghai, 201203 China

**Keywords:** RFC2, Hepatocellular carcinoma, Prognosis, Bioinformatics, Proliferation

## Abstract

**Background:**

Replication factor C (RFC) is closely related to tumor progression and metastasis. However, the functional significance of RFC2 in hepatocellular carcinoma remains unclear.

**Materials and methods:**

In order to solve this problem, the expression of RFC2 in liver cancer patients was analyzed through ONCOMINE, UALCAN, Human Protein Atlas. Survival analysis was conducted using Kaplan–Meier plotter and GEPIA. GO and KEGG enrichment analyses were carried out. The protein–protein interaction (PPI) network was performed through Metascape. Western blotting, cell counting kit-8 and transwell assay were used to detect the effect of RFC2 on cell proliferation and migration.

**Results:**

The transcription and protein level of RFC2 in HCC were overexpressed, which was significantly related to the clinical individual cancer stage and pathological tumor grade of HCC patients. In addition, in patients with liver cancer, higher RFC2 expression was found to be significantly correlated with shorter OS and DFS. Furthermore, the function of RFC2 in liver cancer was DNA replication, and its main mechanism was the phase transition of the cell cycle. Biological experiments demonstrated that knockdown of RFC2 reduced the proliferation and migration of HCC cells.

**Conclusion:**

RFC2 might promote the development of liver cancer, which might be achieved by regulating cell cycle and DNA replication. It could be used as a novel biomarker for the prognosis of liver cancer.

**Supplementary Information:**

The online version contains supplementary material available at 10.1186/s41065-021-00179-9.

## Introduction

Liver cancer is the second leading cause of cancer-related deaths worldwide [1]. Its morbidity and mortality have been on the rise in recent years [2]. Hepatocellular carcinoma (HCC) is the main form of liver cancer (about 90%). Its main risk factors including chronic hepatitis B virus (HBV) and hepatitis C virus (HCV) infection, alcoholism, metabolic diseases, etc., which causes an inflammatory environment in the liver [3]. Long-term inflammatory stimulation leads to chromosomal instability, genetic and epigenetic changes, which may lead to liver cancer [4]. In-depth research on the mechanism of the occurrence and development of liver cancer has been carried out for a long time, however, the molecular events of HCC are not yet fully understood. It is important and urgently needed to identify more valuable biomarkers for early diagnosis and survival prediction.

The latest technology progress and method automation of RNA sequencing make full transcriptome analysis a reality. Previous studies have shown that a large number of genes are differentially expressed in HCC patients, which may become biomarkers for the diagnosis, prognosis and targeted therapy of HCC [5]. The fidelity of DNA replication is usually closely related to cancer progression. DNA damage repair and loss of checkpoint function can lead to genome instability. Replication factor C (RFC) is a primer recognition factor of DNA polymerase, which participates in DNA damage repair and checkpoint control during the cell cycle process [6–8], and is closely related to tumor progression and metastasis [9].

Specifically, the RFC complex recognizes the primer on the template DNA and binds to its end. It can load proliferating cell nuclear antigen (PCNA) onto the DNA template to promote subsequent DNA replication [10]. RFC consists of five subunits, including RFC1-5 [11]. Among them, the RFC2 gene encodes the third largest subunit2 (40 kDa) of the RF-C complex. The cells with RFC2 mutation showed increased the incidence of spontaneous mitosis [12]. Studies have shown that RFC2 was significantly up-regulated in some tumor tissues, such as nasopharyngeal carcinoma (NPC) tissue [13] and choriocarcinoma tissue [14]. Besides, studies have shown that RFC2 was used to predict breast cancer progression and metastasis [15]. These findings suggest that RFC2 might be one of the most important genes regulating cancer. However, the role of RFC2 in the development of HCC is still unknown.

In this study, we analyzed the expression of RFC2 in HCC patients, its relationship with clinical parameters, and survival analysis to solve this problem. In addition, we predicted the function and pathway of 100 similar genes to RFC2.

## Results

### Overexpression of RFC2 in hepatocellular carcinoma

**Fig. 1:**
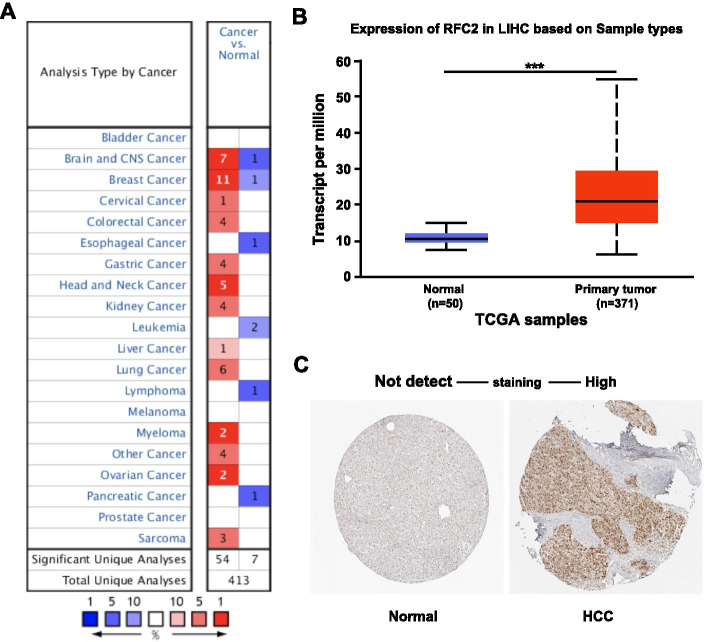
The expression level of RFC2 in HCC. (**a**) The overview of RFC2 mRNA expression in different types of tumors was analyzed using ONCOMINE. (**b**) Transcriptional expression of RFC2 was over-expressed in HCC compared to normal tissues in TCGA through UALCAN. *** *p*<0.001. (**c**) RFC2 protein was not detected in normal liver tissues, however, it was highly expressed in liver cancer tissues

**Fig. 2:**
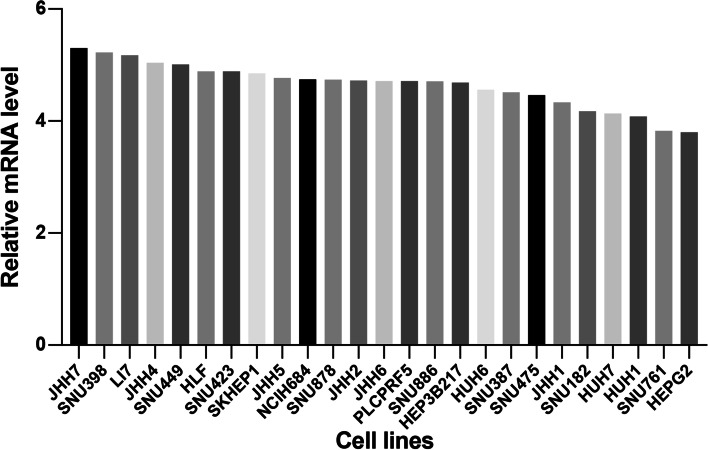
Relative mRNA level in various liver cancer cell lines from CCLE. JHH7 showed the highest RFC2 transcriptional level and HEPG2 showed the lowest

To explore the potential therapeutic value of RFC2 for HCC, ONCOMINE database (www.oncomine.org) and UALCAN database (http://ualcan.path.uab.edu) were used to analyze mRNA expression of RFC2, and Human Protein Atlas (HPA) (https://www.proteinatlas.org/) are used to analyze protein expression. The transcription of RFC2 in various types of cancer was first analyzed (Fig. 1a) and compared with normal tissues through ONCOMINE database. The mRNA expression of RFC2 was found to be markedly higher in liver cancer tissues in the data set. Specifically, in the Roessler Liver 2 dataset [16] (Table 1), RFC2 was overexpressed in HCC tissues compared with normal tissues, with a fold change of 1.645 (*p* = 8.11E-44). Then, the mRNA expression of RFC2 was further measured through UALCAN based on level 3 RNA-seq and clinical data of 31 types of cancer from the Cancer Genome Atlas (TCGA) database, which was different from the ONCOMINE database. The mRNA expression of RFC2 was significantly upregulated in primary liver cancer tissues compared with normal samples (*p* < 0.05) (Fig. 1b). Besides, the protein expression of RFC2 was explored in liver cancer through the Human Protein Atlas (HPA). Immunohistochemistry images displayed that RFC2 protein was not detected in normal liver tissues, however, highly expressed in HCC tissues (Fig. 1c). In general, the transcription and protein of RFC2 are overexpressed in patients with liver cancer. In addition, to find the suitable liver cancer cell line for RFC2 research, the mRNA level of RFC2 in liver cancer cell lines was examined with the CCLE. JHH7 showed the highest RFC2 level and HEPG2 showed the lowest (Fig. 2).

**Table 1:** Significant overexpression of RFC2 mRNA expression between HCC and normal liver tissues in ONCOMINE database

	Types of HCC VS. Liver	Fold Change	*P* value	t-test	Ref
RFC2					
	Hepatocellular Carcinoma	1.645	8.11E-44	16.175	Roessler Liver 2 [15]

### Association of RFC2 with clinical stages and tumor grades of HCC patients

**Fig. 3:**
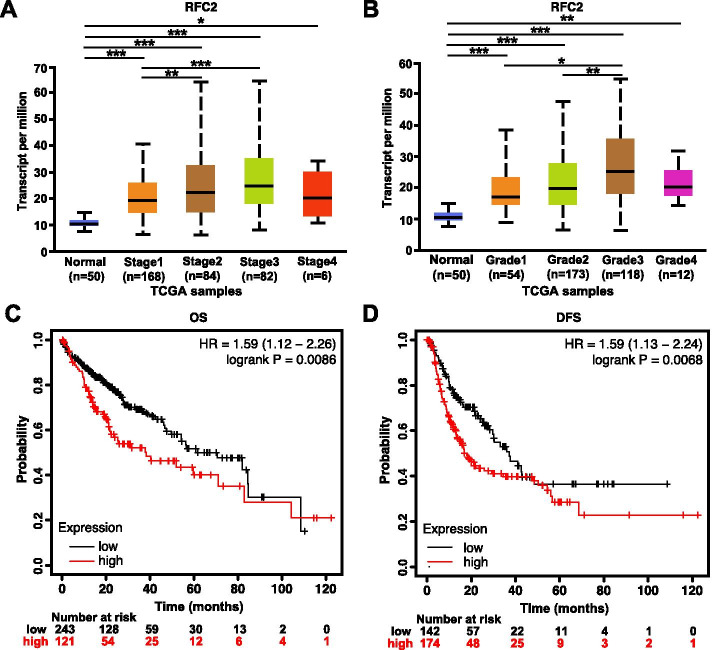
The clinical value of RFC2 expression level in HCC patients. (**a**) RFC2 mRNA expression were significantly related to clinical stages, and as stages increased, the expression of RFC2 tended to be higher. *p<0.05, **p<0.01, ***p<0.001. (**b**) mRNA expression of RFC2 were markedly correlated with HCC grades, and patients with increased grades tended to express higher RFC2 mRNA. *p<0.05, **p<0.01, ***p<0.001. (**c**) Overexpression of RFC2 mRNA were associated with unfavorable OS in HCC. (**d**) Lower expression of RFC2 was correlated with better DFS in liver cancer patients

After the high mRNA and protein expression of RFC2 was found in HCC, the relationship between the mRNA expression levels of RFC2 and clinicopathological parameters was analyzed using UALCAN (http://ualcan.path.uab.edu). As shown in Fig. 3a, the mRNA expression of RFC2 was significantly correlated with the individual cancer stage in HCC, and patients in the advanced stage of tumor tended to express higher mRNA expression of RFC2. The highest RFC2 mRNA expression was found in stage 3. The reason why the mRNA expression of RFC2 in stage 3 seemed to be higher than that in stage 4 might be due to the small sample size (only 6 HCC patients in stage 4). Similarly, as shown in Fig. 3b, the mRNA expression of RFC2 was significantly correlated with tumor grade, and as the tumor grade increased, the mRNA expression of RFC2 tended to be higher. In short, the above results indicated that RFC2 mRNA expression in HCC patients was significantly correlated with clinicopathological parameters.

### Unfavorable effects of elevated RFC2 expression on survival

In addition, the Kaplan–Meier plotter (http://kmplot.com/analysis/) was used to analyze the prognostic information of RFC2 in HCC. As shown in Fig. 3c-d, the mRNA expression of RFC2 was significantly correlated with the prognosis of liver cancer patients. The results indicated that the higher expression of RFC2 mRNA was associated with unfavorable overall survival (OS) (HR = 1.59, 95% CI: 1.12 − 2.26 and *p* = 0.0086), and lower expression of RFC2 (HR = 1.59, 95% CI: 1.13 − 2.24 and *p* = 0.0068) was associated with better disease-free survival (DFS) in patients with liver cancer. These results showed RFC2 might be used as a useful biomarker for predicting the survival of HCC patients.

### Functions and pathways of RFC2 and its similar genes in HCC

**Fig. 4:**
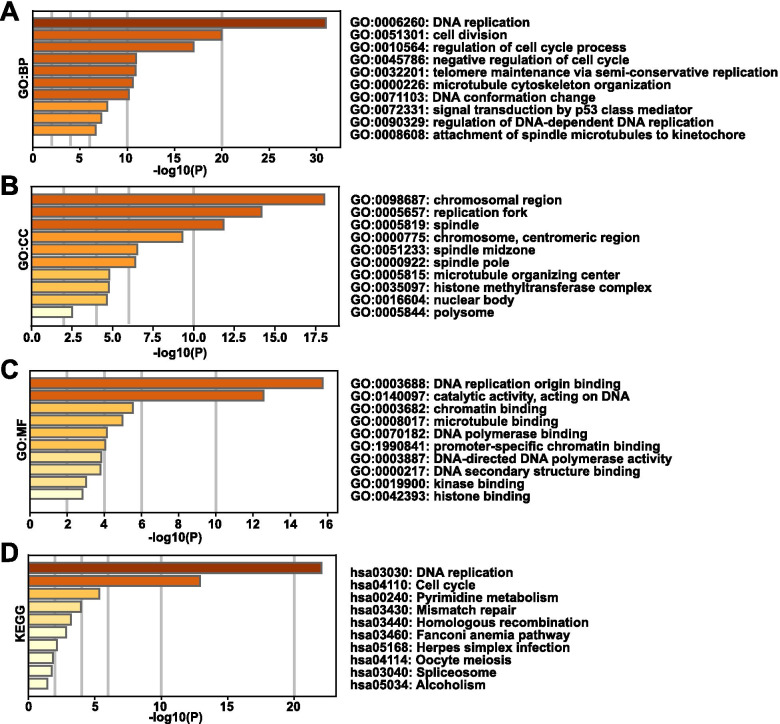
Functions and pathways of RFC2 and its similar genes in HCC. (**a**-**c**) Three main functions of RFC2 and its similar genes were predicted through GO enrichment analysis, including biological process, cellular components and molecular functions. (**d**) KEGG analysis showed the most important pathways of RFC2 and its similar genes in HCC

First, 100 genes similar to RFC2 was found through GEPIA 2.0 (http://gepia2.cancer-pku.cn/#similar). GO and KEGG enrichment analysis was performed for similar genes through Metascape (https://metascape.org/gp/index.html#/main/step1) to deduce possible enrichment functions and pathways. The enriched items in the list of 100 similar genes were shown in Fig. 4. The bar graph corresponded to the P value. Biological processes (BP), including GO:0,006,260 (DNA replication), GO:0,051,301 (cell division), GO:0,010,564 (regulation of cell cycle process) were significantly associated with RFC2 (Fig. 4a). RFC2 also prominently affected the cellular components (CC), such as GO:0,098,687 (chromosomal region), GO:0,005,657 (replication fork), GO:0,005,819 (spindle) (Fig. 4b). In addition, molecular functions (MF) such as GO:0,003,688 (DNA replication origin binding), GO:0,140,097 (catalytic activity, acting on DNA), GO:0,003,682 (chromatin binding) were remarkably regulated by RFC2 in HCC (Fig. 4c). KEGG analysis showed that the most important pathways included hsa03030 (DNA replication), hsa04110 (cell cycle), hsa00240 (pyrimidine metabolism), hsa03430 (mismatch repair) (Fig. 4d).

### Protein–protein interaction analysis

**Fig. 5:**
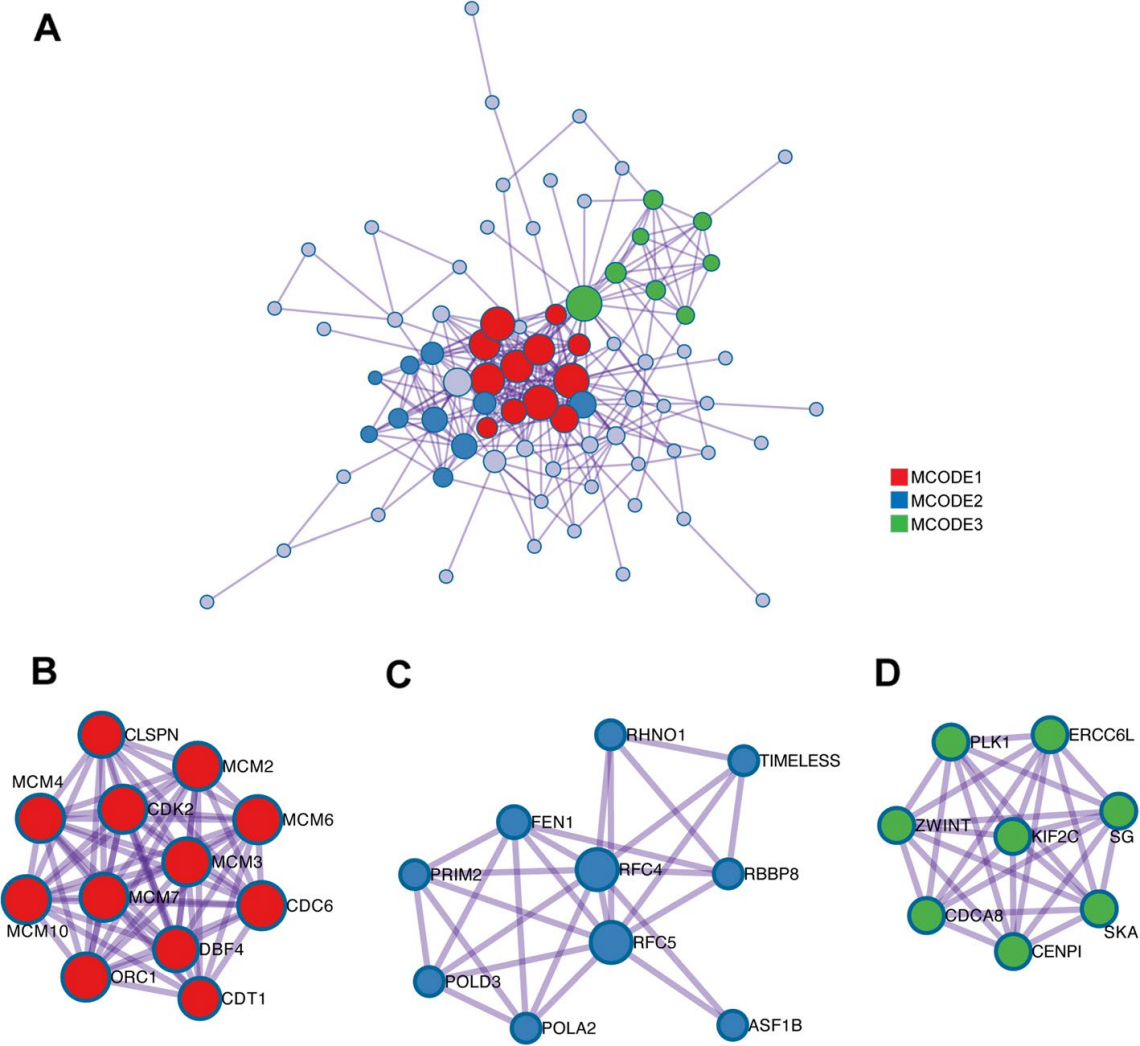
Analysis of RFC2 related proteins based on PPI network. (**a**) Protein-protein interaction network generated using Metascape. (**b**) Mcode1: DNA replication initiation. (**c**) Mcode2: DNA replication. (**d**) Mcode3: chromosome, centromeric region

PPI analysis was performed by Metascape (Fig. 5a). In the input list of 100 similar genes (S1), the main interaction networks were DNA replication initiation (Fig. 5b); DNA replication (Fig. 5c); chromosome, centromeric region (Fig. 5d). In general, DNA replication initiation was the most important interaction network, and the proteins involved mainly included MCM2, MCM3, MCM4, MCM6, MCM7, MCM10, CDC6, CDK2, CDT1, CLSPND, DBF4 and ORC1. In addition, studies have shown that MCM6 in HCC indicates poor tumor characteristics and poor prognosis and promotes cell cycle progression [17], which is consistent with our study.

### Regulation of RFC2 expression on proliferation of HCC cells

**Fig. 6:**
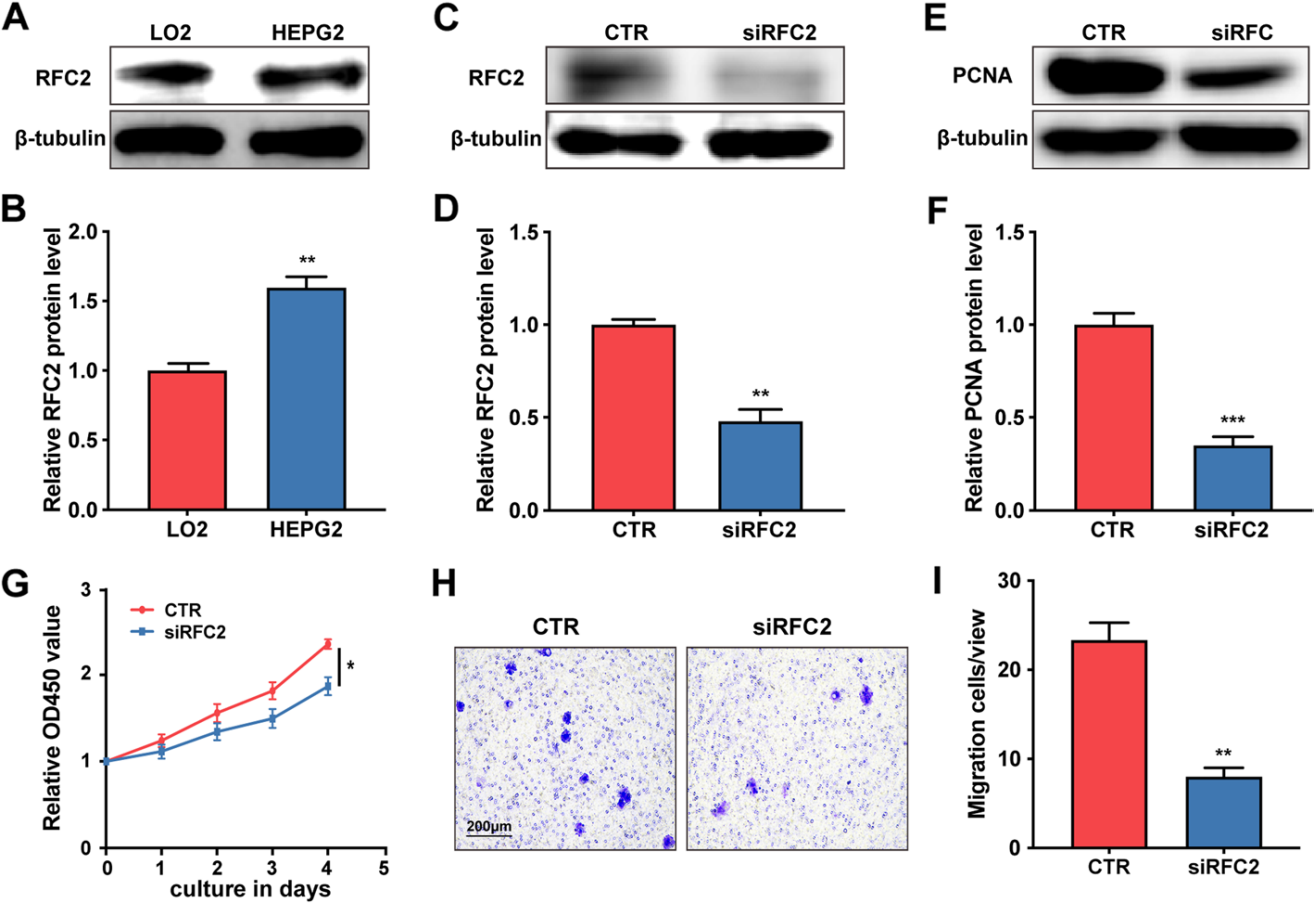
RFC2 promoted the malignant characteristics of HCC cells. (**a**, **b**) Western blotting analysis of RFC2 in LO2 and HEPG2 (**a**) and corresponding protein level quantification (**b**). (**c**, **d**) Western blotting analysis of RFC2 in different groups of HEPG2 (**c**) and corresponding protein level quantification (**d**). (**e**, **f**) Western blotting analysis of PCNA in different groups of HEPG2 (**e**) and corresponding protein level quantification (**f**). (**g**) CCK8 proliferation abilities of HEPG2with different expression pattern of RFC2. (**h**, **i**) Representative images of cell migration by Transwell assay (**h**) and quantification of cells stained with crystal violet in each group (**i**). Scale bar: 200 μm. *P < 0.05, **P < 0.01, ***P < 0.001

Western blotting of RFC2 showed that the level of RFC2 was up-regulated in HCC cells compared with normal hepatocytes (Fig. 6a-b). A functional study was conducted to evaluate the role of RFC2 in the hepatocarcinoma cell line HEPG2. RFC2 was silenced with siRNA (Fig. 6c-d), and its effect on cell proliferation was evaluated by CCK8 analysis. Compared with the control cell line, the OD450 value of the siRFC2 group was significantly lower, indicating that RFC2 regulates the proliferation of HCC cells (Fig. 6g). In addition, the protein expression level of proliferation marker, proliferating cell nuclear antigen (PCNA), were detected by Western blot analysis. As shown in Fig. 6e-f, compared with the control cell line, the expression of PCNA in the siRFC2 group was reduced, which further indicates that RFC2 has a regulatory effect on the proliferation of HCC cells. Transwell assay was used to evaluate the effect of RFC2 on cell migration. Compared with the control group (CTR), siRFC2 inhibited cell migration in the HEPG2 cell line, which was confirmed by the positive staining of crystal violet in the larger purple area of the lower chamber (Fig. 6h-i), implying that RFC2 may affect the metastasis of HCC cells.

## Materials and methods

### Ethics

The research has been approved by the Ethics Committee of the Sixth People's Hospital Affiliated to Shanghai Jiaotong University. All data is from an online database, which has obtained all written informed consent.

### ONCOMINE

ONCOMINE database is a publicly accessible online cancer microarray database (www.oncomine.org) [18]. In this study, the mRNA expression of RFC2 was obtained from the ONCOMINE database between different tumor tissues and their corresponding adjacent normal control samples. The differences in transcription expression were compared through students’ t-test. The specific settings were as follows: fold change: 1.5, P value: 0.01.

### UALCAN

UALCAN (http://ualcan.path.uab.edu) is a user-friendly and comprehensive web resource for analyzing cancer data, which is based on the TCGA database and contains a large amount of clinical data [19]. In this study, UALCAN was used to analyze the relative transcriptional expression of RFC2 in HCC tissues and the relationship between RFC2 and clinicopathological parameters, including clinical stages and HCC grades. The t-test was used to compare the expression of RFC2 in HCC and normal tissues. P value < 0.05 was considered statistically significant.

### Cancer Cell Line Encyclopedia (CCLE)

Cancer Cell Line Encyclopedia (https://portals.broadinstitute.org/ccle) is an online database jointly developed by the Broad Institute and the Novartis Institute of Biomedicine, Novartis Research Foundation, and the Institute of Genomics [20]. This project aims to describe in detail the genetics and pharmacology information of a large number of human cancer models, comprehensive computational analysis that links different pharmacological vulnerabilities and genomic patterns and use cell line integrated genomics to stratify cancer patients. In this study, the mRNA expression data of RFC2 in cancer cell lines was downloaded from CCLE. Then, we screened out the mRNA expression of RFC2 in liver cancer cell lines.

### Human Protein Atlas

The Human Protein Atlas (HPA) (http://www.proteinatlas.org/) uses transcriptomics and proteomics techniques to study protein expression in different human tissues and organs from the RNA and protein levels.

HPA is mainly divided into three sections: Cell, Tissue and Pathology, which respectively show the expression of protein in cells, normal tissues and cancerous tissues [21–23]. This study used immunohistochemical images to compare the protein expression of RFC2 between human normal tissues and HCC tissues.

### Kaplan–Meier plotter

Kaplan Meier Plotter (http://kmplot.com/analysis/) is an online website dedicated to prognostic analysis [24]. In this study, the overall survival (OS) was carried out with Kaplan–Meier plotter analysis, and the prognostic value of the characteristic RFC2 with high expression found in HCC samples was evaluated. The log-rank P was calculated accordingly.

### Gene Expression Profiling Interactive Analysis (GEPIA)

GEPIA (http://gepia2.cancer-pku.cn/#index) is a newly developed interactive web server that analyzes RNA sequencing expression data from TCGA and GTEx projects [25]. In this study, we used GEPIA to find 100 similar genes for RFC2, which were used for subsequent functional analysis.

### Gene Ontology (GO) and Kyoto Encyclopedia of Genes and Genomes (KEGG) analysis

GO and KEGG analyzed the functions and pathways of 100 similar genes in Metascape (http://metascape.org/) [26]. Terms with *p* value < 0.01, minimum count 3 and enrichment factor > 1.5 were collected through Metascape, and they were grouped according to the similarity of their members. GO enrichment analysis predicted the function of RFC2 and 100 similar genes [27]. KEGG analysis predicted the metabolic and regulatory pathways of RFC2 and 100 genes similar to it from the perspective of molecular networks [28].

### Protein–Protein Interaction (PPI) enrichment analysis

The PPI network of 100 similar genes was obtained using the Metascape [26]. For a given gene list (S1), PPI enrichment analysis was performed with the following databases: BioGrid, InWeb_IM, OmniPath [29, 30]. The Molecular Complexity Detection (MCODE) algorithm was further applied to identify densely connected network components if the network contains 3 to 500 proteins. Pathway and process enrichment analysis has been applied to each MCODE component respectively, and the three best score items obtained by P value are used as the function description of corresponding components.

### Cell culture and transfection

LO2 and HepG2 cells (Cell Bank of Chinese Academy of Sciences, Shanghai, China) were maintained in DMEM, 10% FBS (Gibco, Carlsbad, USA) in a humidified condition containing 5% CO_2_ at 37 °C. All cells used in the experiment were passaged 3–5 times. In order to regulate RFC2 expression in vitro, siRNA (RiboBio, Guangzhou, China) was transfected into HepG2 with Lipofectamine®3000 (Invitrogen). When the cells grew to 80% fusion, the original medium was discarded. In short, siRNA and Lipofectamine®3000 were incubated with Opti-MEM (Invitrogen) for 5 min respectively, and then mixed at 25 °C for 10 min. The mixture (with a final concentration of 50 nM siRNA) was added to the cells and replaced with serum medium after 4–6 h. The siRNA sense sequence is CCACAAGCATTCTGTGCTT. SiRNA-control (UAAGGCUAUGAAGAGAUAC) was used as a negative control.

### Western blot

Total protein was harvested using RIPA lysis buffer and PMSF (Beyotime Institute of Biotechnology, Shanghai, China), and centrifuged at 4 °C, 12,000 g for 30 min. Protein concentrations were measured using Bicinchoninic Acid (BCA) Protein Assay Kit (Beyotime Institute of Biotechnology, Shanghai, China). Protein samples were separated by sodium dodecyl sulphate–polyacrylamide gel electrophoresis and then transferred to polyvinylidene difluoride (PVDF) membranes (0.45, Millipore, Billerica, MA, USA). Antibody dilutions were 1: 1000 for the anti-RFC2 antibody, 1: 2000 for the anti-PCNA antibody and 1: 5000 for the anti-GAPDH antibody (Santa Cruz Biotechnology, Santa Cruz, USA). After incubation with primary antibodies, membranes were extensively washed and then incubated with the horseradish peroxidase-conjugated-conjugated secondary antibody (1:1000 diluted, Beyotime Institute of Biotechnology, Shanghai, China) for 1 h at room temperature. The protein bands were visualized by enhanced chemiluminescence substrate Kit (GE Healthcare, NA, UK) and photographed by GE Amersham imager 600 imaging system. β-tubulin (Cell Signaling Technology, Beverly, MA, US) was used as the control.

### Cell counting Kit-8 assay

To detect the proliferation ability of cells, cell viability was detected with Cell Counting Kit-8 every 24 h. HEPG2 cells were seeded in the 96-well plates at a density of 5000 cells per well and grown in an incubator with 5% CO^2^ at 37 ℃. After cell adhesion, each well was replenished with 10 μl cell-counting kit-8 (CCK-8; Yeasen, Shanghai, China) solution and the sample was incubated at 37 °C for 2 h. Finally, the cell optical density (OD) was tested at 450 nm by Molecular Devices Spectra Max i3x. Cell viability (%) was calculated based on the percentage of control. The experiments were carried out in triplicate.

### Transwell assay

Transwell chamber (Costar, Corning, USA) was used for migration. Specifically, cells were seeded into the upper chamber of the Transwell system (1 × 10^5^ cells per well). 600 μl medium containing 10% FBS was added to the lower chamber of the 24 well Transwell system. The cells were then incubated at 37° C for 24 h to migrate. After that, the non-migrating cells in the upper chamber of Transwell system were removed with cotton swabs. The cells on the sub-membranous surface were fixed, stained and photographed, and five random areas of each area were counted under the inverted microscope (Leica, Germany). Image J was used for statistical analysis of the number of migrated cells.

## Discussion

The fidelity of DNA replication is usually closely related to the progression of cancer, including liver cancer. The strict regulation of cell cycle growth signals controls the proliferation of normal cells. DNA damage repair and loss of checkpoint function can lead to genome instability, causing cancer cells to proliferate out of control. RFC is involved in DNA damage repair and checkpoint control during the cell cycle, and has been showed to be an important regulator of the malignant progression of many cancers [13, 14, 31]. Among them, RFC2 has been proven to promote a variety of tumor proliferation [13–15]. However, the biological function of RFC2 in HCC has not been explored. In this study, the expression, function, interaction pathway and prognostic value of RFC2 in HCC were analyzed for the first time to guide the future research of liver cancer. Further functional studies in the HCC cell line showed that knockdown of RFC2 could inhibit proliferation and migration.

Existing studies have shown that RFC2 is significantly upregulated in some tumors, such as nasopharyngeal carcinoma [13], choriocarcinoma [14], and colorectal cancer [32]. Our results found that RFC2 is overexpressed in HCC, which is consistent with previous studies. In addition, the protein expression level of RFC2 was further checked through the HPA database. The immunohistochemical staining intensity of tumor samples is much higher, which is consistent with mRNA expression.

Previous studies have shown that RFC2 is related to the progression and metastasis of breast cancer and can be used as a prognostic indicator for breast cancer patients. CRC patients with higher RFC2 levels showed poor clinicopathological symptom [32]. In this study, the expression of RFC2 mRNA is significantly related to the clinical stage and tumor grade of liver cancer. Patients with advanced cancer and high tumor grade tend to express higher RFC2 mRNA. Further survival analysis showed that higher expression of RFC2 mRNA was associated with poor overall survival (OS) and disease-free survival (DFS).

Previous studies have found that the inhibition of RFC2 can enhance the cytotoxicity of temozolomide to glioblastoma [33]. This article analyzed the functions and pathways of 100 similar genes of RFC2 in HCC patients. We found that DNA replication is the most significant functional enrichment term, which is consistent with our functional findings in vitro. In terms of protein interaction, the PPI network composed of proteins associated with the DNA replication origin was most affected (these proteins included: MCM2, MCM3, MCM4, MCM6, MCM7, MCM10, CDC6, CDK2, CDT1, CLSPND, DBF4 and ORC1).

Some limitations exist in our research. First, all the data in our study comes from online databases. In vivo tests including larger sample sizes are needed to verify the findings in this article and explore the clinical application of RFC2 in the treatment of liver cancer. Secondly, the potential diagnostic and therapeutic role of RFC2 in liver cancer has not been evaluated, so further studies are needed to explore whether RFC2 can be used as a diagnostic marker or therapeutic target. Finally, although the possible pathways are analyzed in this article, further research is needed to explore the direct mechanism of RFC2 on HCC in the future.

In summary, our results show that the transcription and protein of RFC2 in HCC are overexpressed, which is significantly related to the clinical individual cancer stage and pathological tumor grade of HCC patients. In addition, in patients with liver cancer, higher RFC2 expression was found to be significantly correlated with shorter OS and DFS. RFC2 may be a prognostic biomarker for the survival of liver cancer patients. In addition, the function of RFC2 in liver cancer is DNA replication, and the main mechanism is cell cycle phase transition. The JHH7 and HEPG2 cell line may be suitable for the study of the mechanism of RFC2 in liver cancer. This study is the first to study the prognostic correlation of RFC2 in liver cancer, and we believe that the results are worthy of further study.

## Supplementary Information

**Additional file 1.**

## Data Availability

The data used to support the findings of this study are included in the manuscript.

## Abbreviations

HCC: Hepatocellular carcinoma
RFC2: Replication factor C
TCGA: Cancer genome atlas
GO: Gene ontology
KEGG: Kyoto encyclopedia of genes and gnomes
BP: Biological processes
CC: Cellular components
MF: Molecular functions
OS: Over survival
DFS: Disease free survival
CTR: Control
